# Functionalized Fullerene Increases NF-*κ*B Activity and Blocks Genotoxic Effect of Oxidative Stress in Serum-Starving Human Embryo Lung Diploid Fibroblasts

**DOI:** 10.1155/2016/9895245

**Published:** 2016-08-21

**Authors:** E. S. Ershova, V. A. Sergeeva, V. J. Tabakov, L. A. Kameneva, L. N. Porokhovnik, I. I. Voronov, E. A. Khakina, P. A. Troshin, S. I. Kutsev, N. N. Veiko, S. V. Kostyuk

**Affiliations:** ^1^Research Centre for Medical Genetics (RCMG), Moscow 115478, Russia; ^2^V. A. Negovsky Research Institute of General Reanimatology, Moscow 107031, Russia; ^3^Institute for Problems of Chemical Physics of Russian Academy of Sciences, Moscow Region 142432, Russia; ^4^Skolkovo Institute of Science and Technology, Skolkovo Innovation Center, Nobel Street 3, Moscow 143026, Russia; ^5^N. I. Pirogov Russian National Research Medical University, Moscow 117997, Russia

## Abstract

The influence of a water-soluble [60] fullerene derivative containing five residues of 3-phenylpropionic acid and a chlorine addend appended to the carbon cage (F-828) on serum-starving human embryo lung diploid fibroblasts (HELFs) was studied. Serum deprivation evokes oxidative stress in HELFs. Cultivation of serum-starving HELFs in the presence of 0.1–1 *µ*M F-828 significantly decreases the level of free radicals, inhibits autophagy, and represses expression of NOX4 and NRF2 proteins. The activity of NF-*κ*B substantially grows up in contrast to the suppressed NRF2 activity. In the presence of 0.2–0.25 *µ*M F-828, the DSB rate and apoptosis level dramatically decrease. The maximum increase of proliferative activity of the HELFs and maximum activity of NF-*κ*B are observed at these concentration values.* Conclusion*. Under the conditions of oxidative stress evoked by serum deprivation the water-soluble fullerene derivative F-828 used in concentrations of 0.1 to 1 *µ*M strongly stimulates the NF-*κ*B activity and represses the NRF2 activity in HELFs.

## 1. Introduction

Oxidative stress underlies the pathogenesis of some cardiovascular, oncologic, and neurologic diseases. Accumulation of reactive oxygen species (ROS) causes damage to the cellular DNA. In order to minimize the genotoxic effect, novel techniques for ROS reduction are under development.

Over the past decade, nanoparticles based on the molecular carbon form known as fullerenes are considered as promising and efficient ROS scavengers. Fullerenes represent very hydrophobic substances which are virtually not soluble in aqueous media. In order to improve their solubility, various substituents are attached to the carbon cage. Using various model systems, fullerene ability to penetrate through the cell membrane and efficiently reduce the ROS level was unambiguously demonstrated [1–7]. It was revealed that different types of fullerene-based materials are able to intercept all of the major physiologically relevant ROS [8].

It is assumed that fullerenes reduce the ROS level not only due to direct chemical interaction with the reactive oxygen and nitrogen species, but also by influencing the signaling pathways, which regulate the ROS level and help the cells to survive in hostile environment. For example, experiments with polyhydroxylated fullerenes, also known as fullerenols [C_60_(OH)_*n*_], have shown that C_60_(OH)_24_ can attenuate oxidative stress-induced apoptosis* via* augmentation of Nrf2-regulated antioxidant capacity of the cell [9]. Nuclear factor erythroid 2-related factor 2 (NRF2) is a basic leucine zipper redox-sensitive transcriptional factor that plays a key role in ARE- (antioxidant response element-) mediated induction of phase II detoxification and activation of antioxidant enzymes. NRF2 mediates a set of adaptive responses to intrinsic and extrinsic cellular stresses [10]. Fullerene derivative C_60_(C(COOH)_2_)_3_ (C_60_-COOH) was investigated previously and it was demonstrated that C_60_-COOH pretreatment attenuated the lipopolysaccharide-mediated activation of nuclear factor- (NF-) *κ*B and mitogen-activated protein kinase (MAPK) signaling, as well as the production of proinflammatory mediators [11]. Nuclear factor-*κ*B is an essential transcription factor, which controls the expression of genes involved in the immune and inflammatory responses. NF-*κ*B is known to regulate genes involved in apoptosis, cell proliferation, angiogenesis, and metastasis [12, 13]. The signaling pathways, where Nrf2 and NF-*κ*B factors are involved, interact in the cells [14, 15]. It is known that activation of Nrf2-antioxidant signaling attenuates the NF-*κ*B-inflammatory response and elicits apoptosis [14–16]. It suggests that water-soluble fullerenes can act as signaling pathway triggers in the cells, switching on the Nrf2-antioxidant signaling activity and blocking the NF-*κ*B activity. Such behavior of fullerene derivatives might allow their application as therapeutic agents for reducing the genotoxic effect accompanying oxidative stress of different origins and as anti-inflammatory agents.

It was previously shown that serum-free cultivation elicits chronic stress due to increased production of ROS as compared to the serum-supplemented cultures [17]. The increase in the production of free radicals is accompanied by an increase in the population of cells with numerous DSBs. We also observed an increase in the number of cells with condensed and fragmented chromatin due to cultivation in the absence of serum, which is an indicator of ongoing apoptosis. In the stressed, serum-starving fibroblasts, the level and activity of the transcriptional factor NF-*κ*B increase along with elevation of secreted concentrations of TNF*α* cytokine, while the NRF2 activity decreases [17]. Thus, serum-starving HELFs represent a good model to study water-soluble fullerene-mediated NRF2 induction and NF-*κ*B activity repression under the conditions of chronic oxidative stress, which provokes double-strand break formation in the cells. We studied the action of a water-soluble fullerene derivative F-828 carrying COOH groups [18, 19] upon serum-starving human embryo lung diploid fibroblasts (HELFs).

## 2. Methods

Chemical synthesis of a water-soluble fullerene derivative F-828 comprising five residues of 3-phenylpropionic acid and a chlorine atom arranged around one cyclopentadienyl unit on the fullerene cage carrying COOH groups was described by us in detail previously [18, 19].

### 2.1. Cell Culture

Human embryonic lung fibroblasts were obtained from the Research Centre for Medical Genetics RAMS collection. Ethical approval for the use of primary human cells was obtained from the Committee for Medical and Health Research Ethics of Research Centre for Medical Genetics, Russian Academy of Medical Sciences (approval number 5). Before treatments, HELFs were subcultured with 10% serum at most four times. Cells were cultured in growth factor containing serum-free medium “Hybris” [20] that consists of the basal medium and a serum-free supplement containing purified human albumin and a growth factor cocktail (http://www.paneco.ru/). The cells were seeded at 5 × 10^4^/3 mL of medium and then incubated for 2 h to ensure their attachment. Various F-828 concentrations were then introduced and the cells were incubated for 48 h.

### 2.2. Flow Cytometry (FCA)

HELFs were washed in Versene solution and then treated with 0.25% trypsin, washed with culture media, and suspended in PBS. Staining of HELFs with various antibodies was also performed. To fix the cells, they were treated with paraformaldehyde (PFA, Sigma, 2%, 37°C, 10 min). The fixed cells were washed three times with 0.5% BSA-PBS and permeabilized with 0.1% Triton X-100 (PBS, 15 min, 20°C) or with 90% methanol (4°C). Afterwards, the cells were washed three times with 0.5% BSA-PBS and stained with 1 *μ*g/mL antibodies for 2 h (4°C) and then again washed thrice with 0.5% BSA-PBS. Then HELFs were incubated for 2 h (20°C) with the FITC goat anti-rabbit IgG. To quantify DNA, cells were treated with propidium iodide (PI) and RNase A.

The following primary antibodies were used: FITC-*γ*H2AX (pSer139) (Temecula, California); FITC-Ki-67, EEA1, PCNA, BECLIN, NRF2, NF-*κ*B (p65), NF-*κ*B (p65) (pSer529), NOX4, and FITC goat anti-rabbit IgG (Abcam). To quantify the background fluorescence, we stained a portion of the cells with secondary FITC-conjugated antibodies only.

The cells were analyzed using CyFlow Space (Partec, Germany).

### 2.3. ROS Assays

Cells were analyzed using total fluorescence assay in the 96-well plate format at *λ*_ex_ = 488 nm and *λ*_em_ = 528 nm (EnSpire Equipment, Finland). HELFs were treated with 5 *μ*M H_2_DCFH-DA (Molecular Probes/Invitrogen, CA, USA) for 10–60 min at 37°С. The constant of DCF generation rate was determined as a slope of the signal-time line.

### 2.4. Fluorescence Microscopy

The images of the cells were obtained using the AxioScope A1 microscope (Carl Zeiss). Immunocytochemistry: HELFs were fixed in 2% PFA (4°C, 20 min), washed with PBS, and then permeabilized with 0.1% Triton X-100 in PBS (15 min, 20°C), followed by blocking with 0.5% BSA in PBS (1 h, 4°C), and incubated overnight with rabbit polyclonal antibody against LC3 (Epitomics, Cambridge, MA), *γ*H2AX (pSer139), or NF-*κ*B (p65) (Abcam). After washing with 0.01% Triton X-100 in PBS HELFs were incubated for 2 h (20°C) with the FITC goat anti-rabbit IgG, washed with PBS, and then stained with DAPI or PI.

Nuclear fragmentation was examined by Hoechst 33342 (Sigma). Cells were washed and stained with Hoechst 33342 (10 *µ*g/mL) for 10 min at 37°C.

### 2.5. Quantification of mRNA Levels

Total mRNA was isolated using RNeasy Mini kit (Qiagen, Germany). After the treatment with DNAse I, RNA samples were reverse-transcribed by Reverse Transcriptase kit (Sileks, Russia). The expression profiles were obtained using qRT-PCR with SYBR Green PCR Master Mix (Applied Biosystems). The mRNA levels were analyzed using the StepOnePlus (Applied Biosystems); the technical error was approximately 2%. The following primers were used (Sintol, Russia):

 
*BCL2* (F: GCCTTCTTTGAGTTCGGTGG, R: ATCTCCCGGTTGACGCTCT);
 
*BCL2A1* (F: TACAGGCTGGCTCAGGACTAT R: CGCAACATTTTGTAGCACTCTG);
 
*BCL2L1* (F: CGACGAGTTTGAACTGCGGTA R: GGGATGTCAGGTCACTGAATG);
 
*BIRC2* (F: GAATCTGGTTTCAGCTAGTCTGG R: GGTGGGAGATAATGAATGTGCAA);
 
*BIRC3* (F: AAGCTACCTCTCAGCCTACTTT R: CCACTGTTTTCTGTACCCGGA);
 
*KEAP1* (F: GTGGTGTCCATTGAGGGTATCC, R: GCTCAGCGAAGTTGGCGAT);
 
*NFKB1* (F: CAGATGGCCCATACCTTCAAAT, R: CGGAAACGAAATCCTCTCTGTT);
 
*NFE2L2* (F: TCCAGTCAGAAACCAGTGGAT, R: GAATGTCTGCGCCAAAAGCTG);
 
*NOX4* (F: TTGGGGCTAGGATTGTGTCTA; R: GAGTGTTCGGCACATGGGTA);
 
*BAX* (F: CCCGAGAGGTCTTTTTCCGAG, R: CCAGCCCATGATGGTTCTGAT);
 
*TBP* (reference gene) (F: GCCCGAAACGCCGAATAT, R: CCGTGGTTCGTGGCTCTCT). Standard curve method was used for the quantification of RNA levels.

### 2.6. Statistics

All the reported results were reproduced at least three times as independent biological replicates. In flow cytometry, the median of signal intensities was analyzed. The figures show the mean and standard deviation (SD) values. The significance of the observed differences was analyzed with nonparametric Mann-Whitney *U* tests. *p* values < 0.05 were considered statistically significant and marked in figures with (*∗*). Data were analyzed with StatPlus 2007 professional software (http://www.analystsoft.com/).

## 3. Results

**Figure 1:**
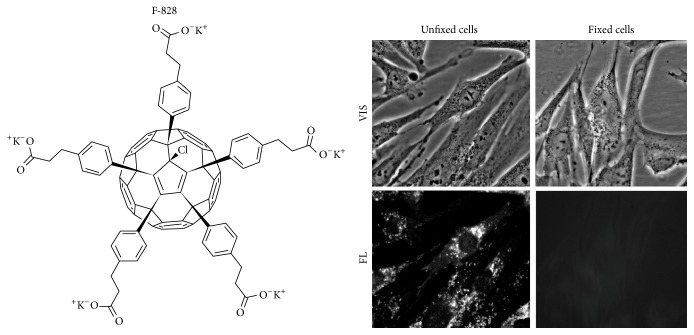
F-828 penetrates through the cell membrane and accumulates in HELFs. Optical microscopy image of HELFs treated with F-828 (0.5 *µ*M). VIS: visible light; FL: fluorescence, *λ*_ex_ = 350 nm. Magnification 40x.

The effects of fullerene on human embryonic lung fibroblasts (HELFs) cultured in serum-free media were studied. We used specially formulated serum-free media “Hybris” containing a growth factor that allows cells to proliferate even in the absence of serum [20]. The molecular structure of the fullerene derivative F-828 comprising five residues of 3-phenylpropionic acid and a chlorine atom arranged around one cyclopentadienyl unit on the fullerene cage is shown in Figure 1. Compound F-828 is well soluble in water and culture medium in the presence as well as in the absence of serum. We used F-828 in concentrations ranging from 0.1 to 2.0 *µ*M for the analysis of its effects on proliferation of HELFs under the conditions of chronic oxidative stress induced by serum deprivation (the model elaborated and reported previously [17]). The fullerene derivative was added to the medium at the initial stage of HELF cultivation, which was as long as 48 hours.

We have revealed that aqueous solutions of this fullerene derivative demonstrate faint dark-red fluorescence when exposed to UV irradiation (300–400 nm). We used this property of F-828 for monitoring of its penetration through membranes and localization in serum-starving HELFs.

### 3.1. F-828 Penetrates through the Cell Membrane and Accumulates in HELFs

The fullerene localization inside the cell was visualized as dark-red fluorescent regions observed under the excitation at a wavelength of 350 nm (Figure 1, unfixed cells). It has been revealed that F-828 penetrates into the cells under our cultivation conditions and gets localized mainly in the area of cytoplasm adjacent to the cell nucleus. Some signals can be also detected in different areas of the cytoplasm. However, no F-828 fluorescence can be detected inside the nucleus. The dark-red fullerene fluorescence can be only observed in unfixed cells, which were analyzed immediately after a short wash-off of the slides with PBS. The signal intensity is considerably reduced when the cells are fixed with 3% paraformaldehyde. The signals completely disappeared after subsequent washing of the fixed cells with 0.1% solution of Triton Х-100 (Figure 1, fixed cells). One can assume that the fullerene nanoparticles interact mainly with the membranes of cells and/or mitochondria. For instance, nanoparticles of soluble fullerene derivative C_60_(OH)_18–22_ were earlier shown to interact with biomembranes [21]. Therefore, degradation of membranes as a result of the formaldehyde fixation and washing them away with a detergent solution also leads to the removal of fullerene nanoparticles from the cells. The revealed property of the F-828 nanoparticles to linger poorly in fixed cells after the cells are rinsed with Triton X-100 enables analyzing the cells with the use of antibodies labeled with fluorochrome, with no need to anticipate artefacts emerging due to fluorescence quenching by the nanoparticles.

### 3.2. F-828 Reduces Endocytosis in HELFs

**Figure 2:**
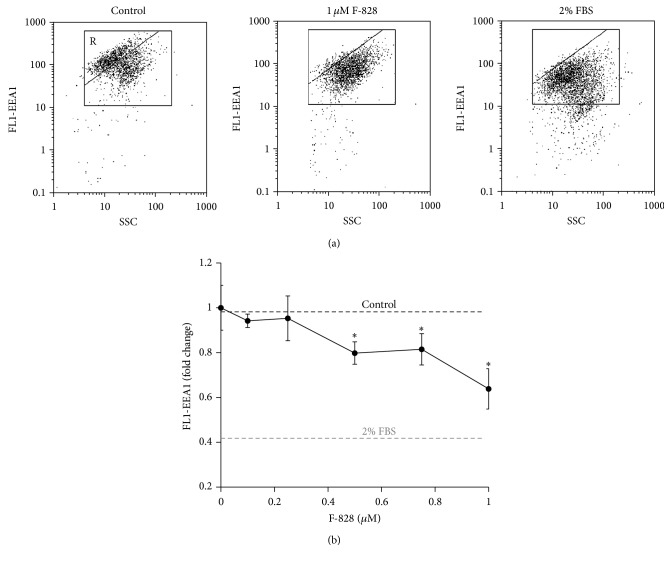
F-828 reduces endocytosis in HELFs. (a) (FCA): the FL1-EEA1 versus SSC plots. Gate R encircles the fraction of HELFs with elevated values of FL1-EEA1. (b) (FCA): dependence of the median values of FL1-EEA1 signals on the F-828 concentration. Concentrations of the fullerene derivative F-828 added to the medium are indicated in the graph.

Endocytosis is one of the common ways of delivery of exogenous compounds into the cell. The formation of novel endosomes is accompanied by an increase in expression of early endosome antigen 1 protein (EEA1), known as an early endosomal biomarker [22]. FITC-labeled antibodies to EEA1 (Figure 2) were used in the assay of early endosome quantity in HELFs using FСA technique. The amount of ЕЕА1 protein increases in the cells under the conditions of serum deprivation. The induced stress leads to intensification of endocytosis. It is notable that approx. 60% of the cells demonstrate upregulated expression of ЕЕА1 (gate R, Figure 2(a)). In the presence of 0.1–1 *µ*M F-828 the percentage of the cells with high level of expression of ЕЕА1 decreases. The mean FL1 signal intensity of the cell is reduced, respectively, by 10–40% (Figure 2(b)).

Thus, the fullerene derivative suppresses endocytosis proportionally to its concentration in the culture medium. Apparently, the fullerene itself does not penetrate the cells by means of endocytosis mechanism. Fullerenes are known to be able to interact efficiently with the cell membranes and to reach the inner membrane layer due to strongly pronounced amphiphilic properties of these compounds [21].

### 3.3. F-828 Has an Effect on HELF Cell Count

**Figure 3:**
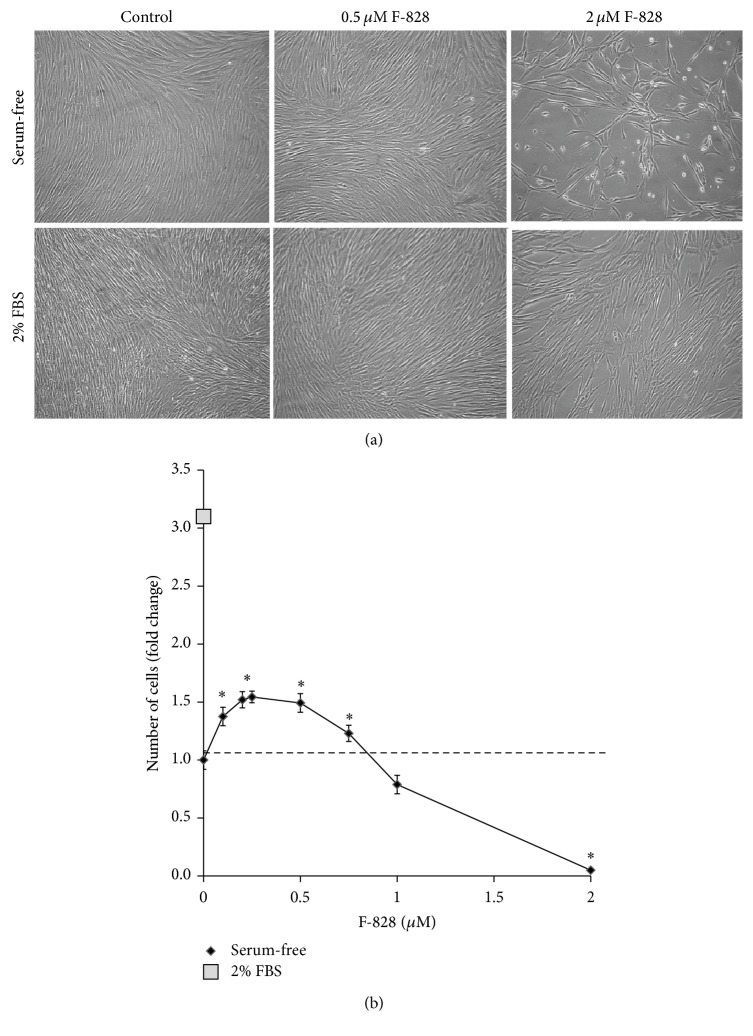
F-828 has an effect on HELF cell count. (a) Microscopy image of HELFs treated with F-828 (magnification 20x). Top row stands for serum-free medium; bottom row shows 2% FBS added. Fullerene concentrations are indicated in the figure. Control means fullerene F-828 was not added. (b) Dependence of the cell count in the culture on the F-828 concentration.

Figure 3(a) shows photographs of HELFs which were cultivated for 48 h in the presence of 2% FBS, in serum-free “Hybris” medium as well as in similar media containing also 0.5 *µ*M and 2.0 *µ*M F-828.  Figure 3(b) presents the dependence of the cell count in the culture on F-828 concentration in the serum-free medium.

The cell count values increased approximately by a factor of 10.3 ± 0.8 in the HELF populations grown in the “Hybris” media supplemented with 2% FBS, while the growth in the serum-free media led to a modest increase by a factor of 3.2 ± 0.3. It is rather remarkable that the number of cells was increased by 50% (*p* < 0.01) and became higher than that of the control experiment when similarly cultured serum-free cells had been exposed to F-828 (0.2–0.5 *µ*M). However, no effects like that were observed in the presence of larger concentrations of F-828 (Figure 3(b)). A notable decrease in the cell counts was observed when using 1.0 *µ*M of F-828. Moreover, the cells virtually did not divide in the presence of 2.0 *µ*M F-828 (Figure 3(a)).

Cell count in culture depends on the rates of cell division and cell death. Therefore, we studied the influence of the fullerene derivative on the cell cycle and death of cells in order to explain the obtained results.

### 3.4. F-828 Affects Proliferative Activity of the HELFs

**Figure 4:**
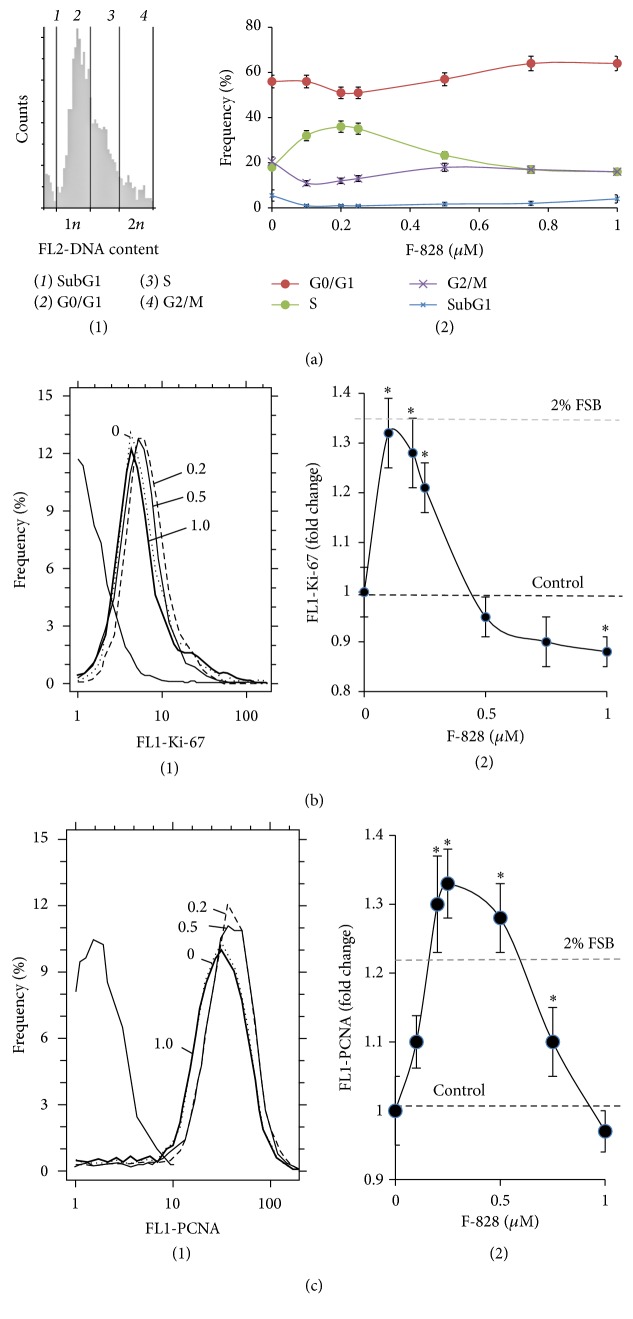
F-828 affects proliferative activity of HELFs. (a) (FCA): (1): distribution of fluorescence intensities of the cells stained with PI; (2): shifts of the proportions of cells with an amount of DNA corresponding to the G1-, S-, and G2/M-phases of cell cycle depending on the F-828 concentration in the culture medium. (b) (FCA): (1): distribution of the cells treated with F-828 according to the FL1-Ki-67 signal strength; (2): dependence of the median values of the FL1-Ki-67 signals on the fullerene concentration. (c) (FCA): (1): distribution of the cells treated with F-828 according to the FL1-PCNA signal strength; (2): dependence of the median values of FL1-PCNA signals on the F-828 concentration.

The cell cycle-related effects induced by F-828 were studied in HELFs that were harvested 48 hours after addition of the fullerene derivative to the media (Figure 4). The cells were stained with antibodies specific to the proliferation markers Ki-67 and PCNA [23, 24] and enumerated by FCA. Additionally, the cell populations were also counted after DNA-specific propidium iodide (PI) treatment.  Figure 4(b) shows the distribution of the cells with various Ki-67 contents. The serum starvation leads to a decrease in the proliferating cell fraction as compared to the FBS-supplemented control cells (Figure 4(b)). When serum-free HELFs were exposed to 0.1–0.25 *µ*M F-828, the fractions of Ki-67-positive cells were increased as compared to the serum-free HELF controls. On the contrary, introduction of 0.5–1.0 *µ*M of F-828 resulted in a decrease in the size of the Ki-67-expressing cell fractions in serum-free HELFs.

A similar analysis was performed for PCNA. When cultivating culture medium was augmented with 2% FBS, the proportion of PCNA-positive proliferating cells increased as compared to the cultures grown in serum-free media. When the cells were exposed to 0.1–0.75 *µ*M of F-828, the proportions of PCNA-positive cells were increased as compared to the serum-free HELF controls (Figure 4(c)). An increase in the F-828 concentration resulted in less pronounced effects. Interestingly, the proportion of PCNA-positive cells increased when the cells were exposed to 0.5 and 0.75 *µ*M of F-828, thus producing an overall picture, which is substantially different from that of Ki-67 staining (Figure 4(b)).

The increase in the HELF proliferative activity in the presence of small amounts of F-828 is confirmed by the data on the DNA content in the cells (Figure 4(a)). Fullerene derivative F-828 used in concentrations of 0.1–0.25 *µ*M induced an increase in the number of cells in the S-phase (*p* < 0.01). The ratio of the cells in the G0/G1 cycle phase decreases (*p* < 0.05).

Propidium iodide staining for DNA content has revealed that HELF population grown in serum-free media shows an increased contribution from the G2/M cells (23% versus 7% for the medium with 2% FBS), Figure 4(a). An exposure of the cells to 0.1–0.25 *µ*M F-828 leads to a decrease in the number of G2/M cells by ~50%. This fact suggests that low fullerene concentrations lead to cancelling the G2/M arrest of a considerable fraction of cells caused by cultivation under the specified stress conditions. The cells exposed to 0.5–1.0 *µ*M F-828 show similar proportions of the G2/M cells to those of control serum-free cells.

### 3.5. F-828 Shifts the Levels of Autophagy and Death of the HELFs

**Figure 5:**
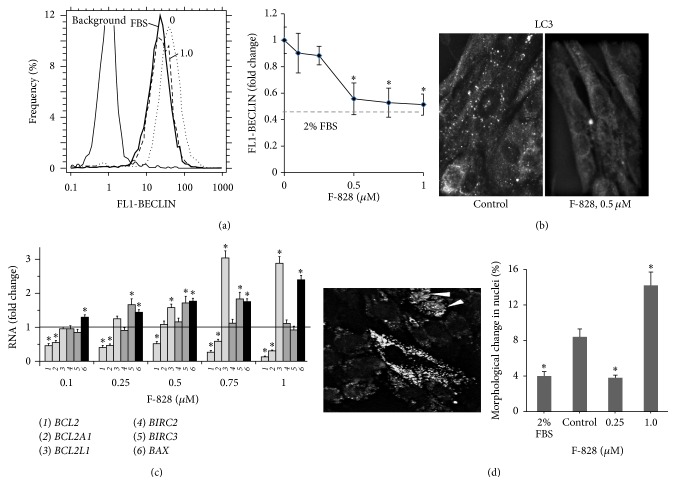
F-828 shifts the levels of autophagy and mortality of HELFs. (a) (FCA): distribution of the cells treated with F-828 according to the FL1-BECLIN signal strength; dependence of the median values of FL1-BECLIN signals on the F-828 concentration. (b) (Fluorescence microscopy): changes in the levels of LC3-positive puncta in the HELFs cells treated with 0.5 *µ*M of F-828. (c) (qRT-PCR): changes in the levels of mRNAs encoding BCL2A1, BCL2, BCL2L1, BIRC2, BIRC3, and BAX in HELFs. (d) (Fluorescence microscopy): morphological changes in the HELFs nuclei. Cells were stained with Hoechst 33342. Photo: HELFs in serum-free media (1 *µ*M of F-828). Arrows point towards the cells, whose nuclei were entered by Hoechst 33342. The histogram presents the proportion of the cells with modified nuclei.

Low cellular nutrient levels can activate autophagy, which acts to restore metabolic homeostasis through the degradation of macromolecules to provide nutrients. Oxidative stress can boost the autophagy considerably. Autophagy, in turn, contributes to the reduction of oxidative damage by consuming the oxidized substances with their subsequent degradation [25]. Under the conditions of serum starvation stress HELFs demonstrate a significant activation of autophagy. A protein known as beclin is one of the autophagy markers. In order to perform an assay of this protein, antibodies to beclin and FAC technique were used. The quantity of beclin in the cells cultivated in the serum-starving medium is increased by a factor of 2 as compared to the cells cultivated in the presence of 2% FBS (Figure 5(a)). The introduction of the fullerene derivative to the culture medium in concentrations of 0.1–1.0 *µ*M leads to a decrease in the amount of beclin in the cells by a factor of 1.1–2.0, respectively. We have also detected autophagosomes (puncta) in the cytoplasm after immunolabeling with the rabbit polyclonal antibody against LC3 [26]. The introduction of F-828 to the culture medium at a concentration of 0.5 *µ*M leads to a decrease in the amount of puncta in the cytoplasm of serum-starving HELFs (Figure 5(b)). Apparently, F-828 suppresses autophagy in the serum-starving HELFs.

To evaluate DNA damage degree in treated and control cells, we quantified the number of cells in the subG1 phase (Figure 4(a)(2)). Under the stress conditions the subG1 cell fraction was increased by a factor of 2 compared to the control cells cultivated in the presence of 2% of serum (8% versus 4%). The cells exposed to 0.1–0.25 *µ*M F-828 showed decreased contents of the subG1 fraction in comparison with the control serum-free cells. The cell cultures exposed to 1.0 *µ*M F-828 did not demonstrate further decrease in the subG1 cell fraction.

We also investigated the appearance of cells exhibiting nuclear chromatin condensation using fluorescent microscopy in order to evaluate the damage degree in treated and control HELFs (Figure 5(d)). The Hoechst 33342 staining revealed that approx. 10% of the cells grown in the serum-free medium show typical morphological hallmarks of apoptosis, including nuclear chromatin condensation. The percentage of the damaged nuclei decreased when HELFs were exposed to 0.25 *µ*M F-828. An exposure of the cells to 1.0 *µ*M F-828 produced an opposite effect characterized by an elevated content of the damaged nuclei.

We analyzed changes in the expression patterns of five genes belonging to BIRC and BCL families, which participate in the antiapoptotic cell response (Figure 5(b)), and also* BAX* gene involved in the apoptosis induction. It was revealed that the level of the* BAX* mRNA increased in the presence of F-828. F-828 led to decreased expression of the antiapoptotic genes* BCL2* and* BCL2A1 *in the cells. In parallel, the level of expression of the three other antiapoptotic genes,* BCL2L1*,* BIRC2*, and* BIRC3*, remains the same or becomes increased. The fact of different regulation of the expression of antiapoptotic genes in the presence of fullerene requires a special study.

### 3.6. F-828 Reduces the ROS Level in Serum-Starving HELFs

**Figure 6:**
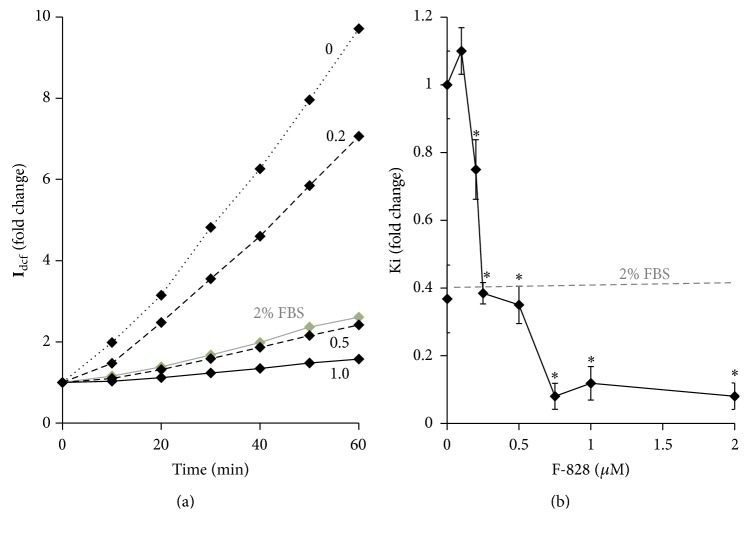
F-828 reduces the ROS level in serum-starving HELFs. (a) (Plate reader): the change of DCF fluorescence depending on the duration of the cell incubation in the presence of 10 *μ*M of H2DCFH-DA. (b) Dependence of the DCF synthesis rate constant in serum-starving HELFs on F-828 concentration. A square denotes DCF synthesis rate in the presence of 2% FBS.

Cultivation of HELFs in a serum-starving medium is known to result in a notable increase in the ROS level (Figure 6(a) and [17]). To study the possible influence of the fullerene on the intracellular ROS levels, the ROS were measured using dichlorodihydrofluorescein diacetate (H2DCFH-DA) dye. The experiment was carried out using a plate reader with a thermocontrolled platform. Figure 6(a) shows the results of the ROS assay performed on living cells.  Figure 6(b) indicates relative values of the constants determined from the slopes of the straight lines that approximate dependence of DCF fluorescence intensity versus time (Figure 6(a)).

Fullerene derivative F-828 added at 0.2–2.0 *µ*M suppresses the ROS formation in the cells. One can notice two discrete stair-like steps on the curve showing the dependence of the DCF formation rate on the fullerene concentration. When the fullerene concentration increases from 0.1 up to 0.25 *µ*M, the reaction rate decreases by 60% and then stays almost constant in the range of concentrations between 0.25 and 0.5 *µ*M. An increase in the fullerene concentration up to 0.75 *µ*M decreases further the DCF production rate by more than 30%.

There are several possible mechanisms, which can be responsible for the observed decrease in the ROS level in the presence of fullerene derivative. For instance, the fullerene derivative can harvest ROS directly* via* radical addition pathway. It is also possible that the fullerene derivative influences the enzymes and transcription factors responsible for ROS production and disposal in the cell.

### 3.7. F-828 Causes a Decrease in the Level of NOX4 Protein in Serum-Starving HELFs

It has been shown that production of cellular ROS is related to the action of NAD(P)H-oxidase type of enzymes, predominantly those encoded by NOX gene family [27]. NAD(P)H-oxidase 4 (NOX4) has been recognized recently as a major source of ROS in HELFs and it was shown to be implicated in the fibrogenic response to lung injury [28]. In living cells, NOX4 catalyzes the reaction responsible for the hydrogen peroxide formation.

**Figure 7:**
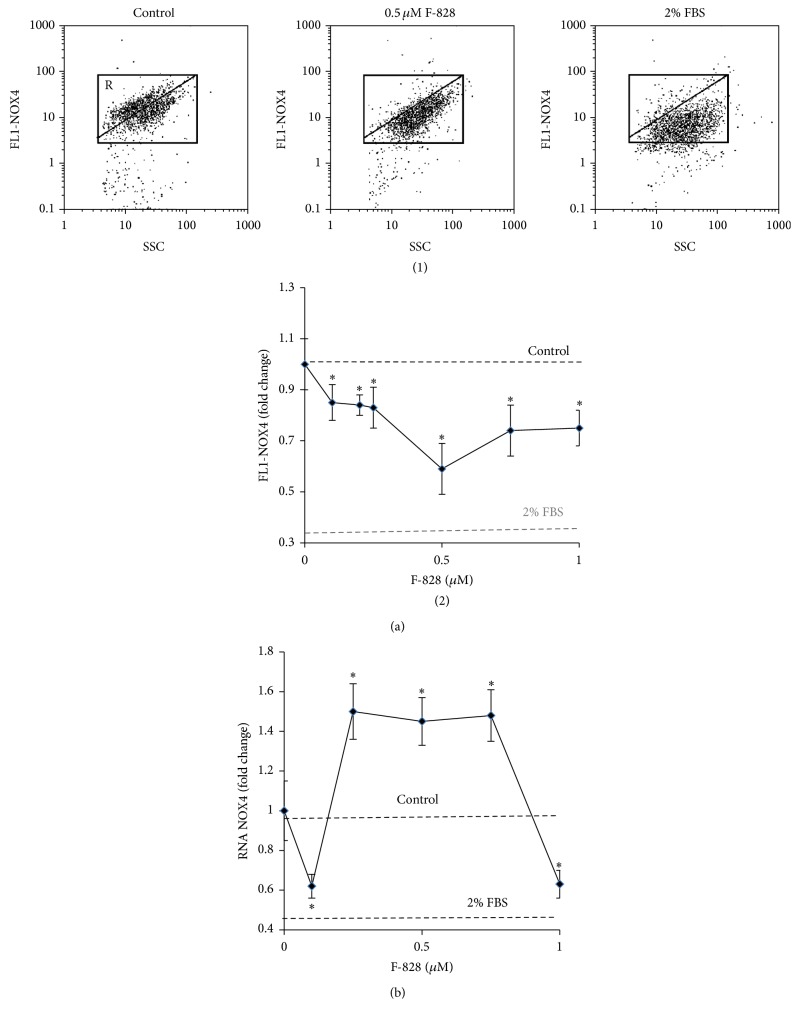
F-828 entails a decrease in the level of NOX4 protein in serum-starving HELFs. (a) (FCA): (1): the FL1-NOX4 versus SSC plots. Gate R encircles the fraction of HELFs with elevated values of FL1-NOX4; (2): dependence of the median values of the FL1-NOX4 signals on the fullerene concentration. (b) (qRT-PCR): changes in the levels of mRNAs encoding* NOX4* in HELFs.

The level of NOX4 protein was determined in HELFs using FCA and antibodies specific to NOX4 (Figure 7). The population of serum-starving HELFs comprises two cell fractions: one with elevated NOX4 (gate R on the plot of FL1-NOX4 versus SSC) representing about 60% of the total amount of the cells and the other with a lower NOX4 content. For comparison, HELFs cultivated in the presence of 2% FBS contain just 7% of cells with high level of NOX4 protein.

The mean level of NOX4 protein in HELFs cultivated under the serum starvation conditions is 3 times higher than that in the cells grown in the medium containing 2% of FBS (Figure 7(a)). Interestingly, the rate of DCF production in the serum-starved cells also appeared to be 3 times higher than in the control cells, which were cultivated in the presence of 2% of serum (Figure 6).

The addition of F-828 to the serum-free medium in concentrations of 0.1–1.0 *µ*M induced a notable decrease in the content of NOX4 protein in the cells. The most prominent response was observed in the presence of 0.5 *µ*M of F-828 when the average intensity of FL1-NOX4 decreased by 60% and the fraction of cells with high NOX4 level (gate R) decreased down to a remarkable value of 2%.

We also analyzed the evolution in the content of* NOX4* mRNA in serum-starving HELFs (Figure 7(b)). It has been revealed that addition of the fullerene derivative to the medium led to an increase in the* NOX4* mRNA content by a factor of 1.4–1.6 at any of the studied F-828 concentrations except for 0.1 and 1 *µ*M. This observation suggests that* NOX4* gene expression is regulated presumably at the posttranscriptional level in the fullerene-treated cells.

Finally, we can emphasize that the revealed strong reduction of the content of NOX4 protein in the cells is one of the key factors responsible for the decrease of ROS in serum-starving HELFs in the presence of fullerene.

### 3.8. F-828 Reduces NRF2 in Serum-Starving HELFs

**Figure 8:**
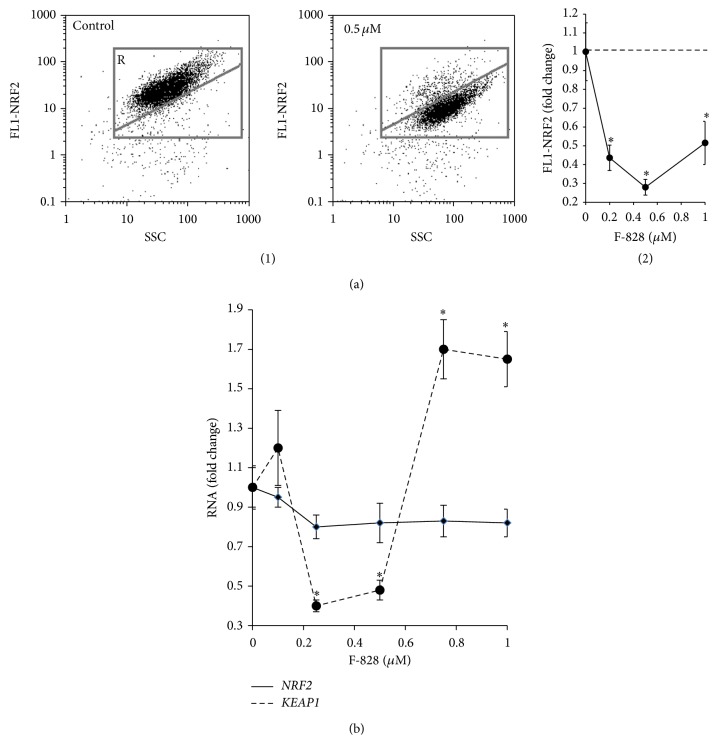
F-828 reduces NRF2 in serum-starving HELFs. (a) (FCA): (1): the FL1-NRF2 versus SSC plots. Gate R encircles the fraction of HELFs with elevated values of FL1-NRF2; (2): dependence of the median values of FL1-NRF2 signals on the fullerene concentrations. (b) (qRT-PCR): changes in the levels of mRNAs encoding* NRF2* and* KEAP1* in HELFs.

The NF-E2 related factor 2 (NRF2) regulates constitutive and inducible expression of ARE-driven genes through a dynamic pathway involving nucleocytoplasmic shuttling [29, 30]. NRF2 controls ROS production involving mitochondria and NADPH oxidase [31]. NOX4-NRF2 imbalance is considered as an origin of pathological fibrosis [28]. NRF2 factor is expressed in serum-starving HELFs at the same level as in the cells growing in the presence of 2% of serum. However, this factor is not active and it is localized only in cytoplasm of the serum-starving HELFs [17]. In the cells cultivated in presence of serum NRF2 is located in the nucleus. This fact indicates an activation of NRF2 [17]. The content of NRF2 was measured using FCA and antibodies specific to NRF2 (Figure 8).

We observed that cultivating HELFs in the serum-starving medium in the presence of fullerene leads to a significant decline of NRF2 protein level in the cells (Figure 8(a)). Using fluorescence microscopy, it was shown that NRF2 is still located solely in the cell cytoplasm; that is, it is inactive (data not shown). The observed lowering of the NRF2 protein content in the serum-starving HELFs in the presence of fullerene could be considered as a consequence of a considerable reduction in the level of ROS and NOX4 protein. It was previously shown that intracellular NOX4 reduction was accompanied by a decrease in the amount of NRF2 transcription factor [32, 33].

We also analyzed the quantity of mRNA encoding NRF2 protein in serum-starving HELFs (Figure 8(b)). The introduction of fullerene derivative to the medium resulted in a slight decrease in* NRF2* mRNA in the whole range of F-828 concentrations involved in our experiments. Presumably, the regulation of* NRF2* gene expression, as well as that of* NOX4* gene, occurs at some posttranscriptional level in serum-starving cells treated with the fullerene derivative. NRF2 is known to be negatively regulated by cytoplasmic Kelch-like ECH-associated protein 1 (Keap1). We observed an increase in the concentration of mRNA for KEAP1 protein at 0.75 and 1 *µ*M of F-828.

Thus, NRF2 transcription factor is not active in the serum-starving HELFs, which were cultivated in the presence of fullerene derivative. Therefore, NRF2 is neither a cause nor a factor of the observed significant decrease in ROS level in serum-starving HELFs cultivated in the presence of fullerene.

### 3.9. F-828 Affects the Content of Phosphorylated Form of H2AX Histone in Serum-Starving HELFs

One of the methods of revealing the DNA DSBs is based on the fact that a highly conservative histone protein (H2AX) is phosphorylated at the residue of serine 139 in the site where the development of DNA break starts with the participation of ATM, ATR, and DNA-PK kinases [34]. The reaction rapidly propagates engaging hundreds to thousands of molecules of H2AX, which can comprise up to several megabases of the chromatin DNA flanking the DSB site. Bounds with the labeled antibodies and phosphorylated histones called *γ*H2AX foci are available for visualization in the cellular nucleus; their accumulation in great amounts indicates the beginning of the development of apoptosis in the cellular population.

**Figure 9:**
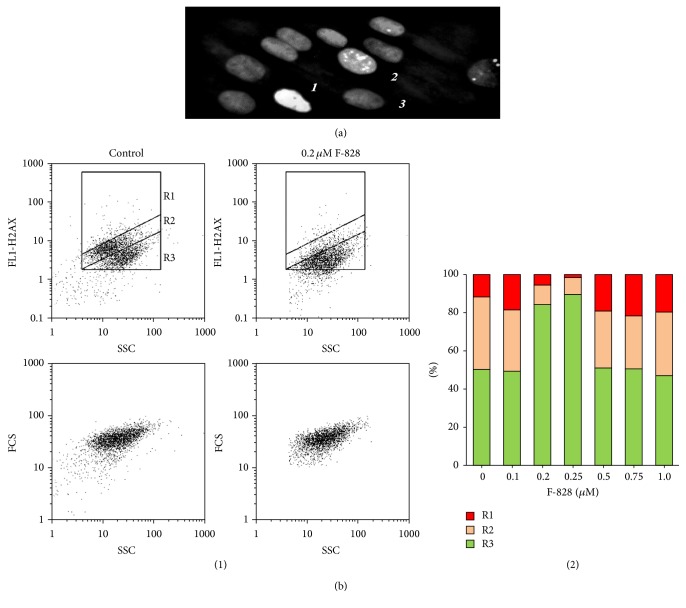
F-828 shifts the content of phosphorylated form of H2AX histone in serum-starving HELFs. (a) (Fluorescence microscopy, ×40): cells were processed for immunofluorescence staining with anti-*γ*H2AX antibody and PI. Detected types of nuclei are denoted with digits: 1: nucleus with multiple dsDNA breaks; 2: nucleus with a few dsDNA breaks; 3: nucleus without gamma-foci. (b) (FCA): (1): the FL1-*γ*H2AX versus SSC plots. Gates R1, R2, and R3 encircle the fractions of HELFs with various content of *γ*H2AX. (2): changes of the proportions of three cell fractions in the HELF cell pool depending on the F-828 concentration.

The image of the fixed serum-starving HELFs stained with FITC-labeled antibodies specific to *γ*H2AX is shown in Figure 9(a). The performed analysis revealed three types of cells: those without any label (3, Figure 9(a)), cells with small quantity of *γ*H2AX (2), and those with a large number of overlapping spots due to the labeled *γ*H2AX (1). FCA procedure (Figure 9(b)) also allowed us to find areas corresponding to these three cell fractions on the FL1 (*γ*H2AX), SSC plot. R1 fraction (12% of the entire population) is represented by the cells with a high content of *γ*H2AX, R2 fraction (39%) corresponds to the cells with a lower *γ*H2AX content, and R3 fraction represents the cells, which have no *γ*H2AX (51%). Thus, almost a half of serum-starving HELFs express detectable amounts of *γ*H2AX.

The R1 fraction consists of the cells with a very high gamma-focus level. Apparently, they are cells undergoing apoptosis. In the presence of serum, the R1 fraction size reduces significantly from 12% down to 4%.

We registered elevation of the average number of *γ*H2AX in the cells by a factor of 1.8–2.2 in the presence of 0.1, 0.5, 0.75, and 1.0 *µ*M of the fullerene derivative (Figure 9(b)(2)). When the concentration of F-828 was equal to 0.2 or 0.25 *µ*M, the average number of *γ*H2AX in the cells was decreased by a factor of 1.5–2.0. One can notice in the histogram shown in Figure 9(b) that the cell counts corresponding to R1 and R2 fractions considerably decreased.

### 3.10. Exposure of the Cells to F-828 Stimulates an Increase in the Activity of NF-*κ*B Factor

HELFs express relatively low amounts of NF-*κ*B when they are grown in the presence of 2% serum [17]. The factor is inactive and located solely in cytoplasm. It can be assumed that serum contains some molecule(s) that strongly inhibit(s) NF-*κ*B activation [35]. In serum-starving cells, the level of NF-*κ*B (p65) increases to a substantial degree along with the translocation of NF-*κ*B (p65) closer to the nucleus [17, 35]. Thus, the serum-starving stress induces the NF-*κ*B activity in the cells. It is well known that NF-*κ*B (p65) is activated by phosphorylation, which plays a key role in the regulation of its transcriptional activity and is associated with nuclear translocation of the factor [36]. Flow cytometry was applied for quantification of HELFs that contain phosphorylated Ser529 (p65) [17]. It has been shown that serum-starving entails a substantial increase in the proportion of the cells that contain phosphorylated Ser529 in p65, thus confirming that the transcriptional factor is active in these cells.

**Figure 10:**
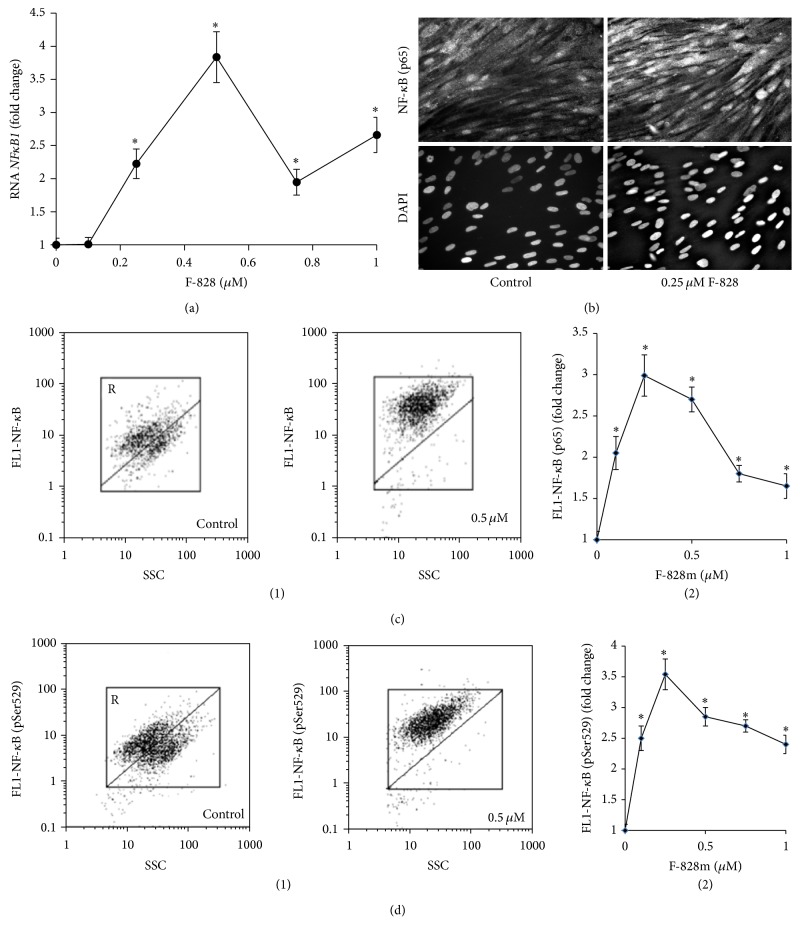
F-828 promotes an increase in the activity of NF-*κ*B. (a) (qRT-PCR): changes in the levels of mRNAs encoding* NFκB1* in HELFs. (b) (Fluorescent microscopy): photo of the cells stained with anti-p65 (FITC) antibodies and DAPI, ×40. (c) (FCA): (1): the FL1-NF-*κ*B (p65) versus SSC plots. Gate R encircles the fraction of HELFs with elevated values of FL1-NF-*κ*B (p65). (2): dependence of the median values of FL1-NF-*κ*B (p65) signals on the fullerene concentration. (d) (FCA): (1): the FL1-NF-*κ*B (pSer529) versus SSC plots. Gate R encircles the fraction of HELFs with elevated values of FL1-NF-*κ*B (pSer529). (2): median values of the FL1-NF-*κ*B (pSer529) signals as a function of the fullerene concentration.

The introduction of the fullerene derivative F-828 in concentrations of 0.1–1.0 *µ*M provokes a 2- to 3-fold increase of total p65 protein amount, while the content of its phosphorylated form Ser529 (p65) becomes 2.5 to 3.5 times higher. The maximum effect of F-828 was observed at a concentration of 0.25 *µ*M (Figures 10(c) and 10(d)).

The elevation of р65 protein is also confirmed by fluorescence microscopy (Figure 10(b)). The fluorescence intensity of HELFs nuclei stained with antibodies specific to р65 gets considerably increased when the cells are cultivated in the presence of fullerene. It is notable that the protein in this case is located only in the cell nuclei.

## 4. Discussion

Here we studied the influence of water-soluble fullerene F-828 on human embryo lung fibroblasts cultivated under the conditions of chronic oxidative stress. The oxidative stress in HELFs was induced by serum deprivation. It is known that cell cultivation in a serum-starving medium drastically boosts the ROS production, particularly in mitochondria [37].

Previously, we described in detail the changes occurring in the HELFs cell culture under serum deprivation conditions [17]. Deficiency of nutrients in the environment slows down the cell proliferation and increases the proportions of SubG1/G0, G2/M, and apoptotic cells. Additionally, the number of double-strand DNA breaks grows up considerably in the cells. Intensification of endocytosis is observed in the cells as a response towards low-nutrient environment conditions. Elevated endocytosis is accompanied by a high level of ЕЕА1 protein, which is a marker of early endosomes (Figure 2).

The induction of oxidative stress is promoted by increased NOX4 gene expression leading to few times higher concentrations of the corresponding protein than those in the control cells (Figure 7). At the same time, the activity of transcription factor NRF2 is blocked. The level of this protein, which is the master switch of the cellular antioxidant response, is decreased, and moreover all the protein is located in cytoplasm. In the control cell culture cultivated in the presence of 2% serum, this factor is active and located in the cell nuclei [17]. An imbalance between the elevated NOX4 gene expression and blocked NRF2 activity is a specific feature of this model system.

Oxidative stress can also stimulate autophagy, which is a cellular process used to recycle the cytoplasmic components. Certain cellular stresses including nutrient depletion are known to induce autophagy [38]. Autophagy induced in the serum-starving HELFs is reflected in a twice higher concentration of autophagy marker Beclin 1 and higher number of LC3 puncta than in the control cells (Figures 5(a) and 5(b)). Recently, autophagy and Nrf2 were shown to be interconnected* via* a direct interaction between p62 (an autophagy adaptor protein) and Keap1 (the Nrf2 substrate adaptor for the Cul3 E3 ubiquitin ligase). Dysregulation of autophagy was shown to result in prolonged Nrf2 activation in a p62-dependent manner [39]. In the case of the investigated system (serum-starving HELFs), the elevated autophagy was followed by the expected decrease in the NRF2 activity.

Serum-starving stress is known to enhance the NF-*κ*B activity in the cells [17, 35]. Activation of NF-*κ*B can prevent cell death and promote cell growth. We also found that the serum-starving HELFs demonstrated elevated contents of total protein р65 and its active phosphorylated form compared with HELFs cultivated in the presence of 2% FBS. The observed localization of NF-*κ*B in cell nuclei confirms its activation. It was revealed previously that NF-*κ*B- and Nrf2-pathways can intersect [14–16]. Generally, an activation of either factor entails blocking the activity of the other one. Indeed, the factor NF-*κ*B is considerably activated, while the activity of NRF2 is blocked in serum-starving HELFs.

Considering the chemical structures of С_60_ fullerene one can assume that this compound might demonstrate high antioxidant capacity. The ability of the fullerene to bind and neutralize ROS directly can lead to the reduction of the ROS level in the serum-starving HELFs and improvement of cell culture's viability. Indeed, serum-starving HELFs appeared to be very sensitive to the low fullerene concentrations (0.2 to 1.0 *µ*M), which indeed decreased the ROS level remarkably. The most pronounced effects were observed at a F-828 concentration above 0.5 *µ*M (Figure 6). It is rather remarkable that the fullerene derivative not only binds ROS directly, but also affects signaling pathways involved in ROS production and scavenging. In particular, introduction of F-828 reduced the level of the NOX4 protein (Figure 7(a)).

The fullerene derivative reduced even to a greater extent the content of NRF2 factor, which is the master switch of antioxidant response (Figure 8(a)). These findings are very distinct from the previous report where a mixture of water-soluble fullerenols was shown to increase the activity of NRF2 factor [9]. It was assumed that C_60_(OH)_24_ attenuates oxidative stress-induced apoptosis* via* augmentation of Nrf2-regulated cellular antioxidant capacity [9]. We have also shown that a decrease in the NRF2 activity is accompanied by reduced autophagy as concluded from the evolution of the Beclin 1 and LC3 puncta content in the cells. This is rather unexpectable result, which warrants further investigations. It was shown previously that suppression of autophagy under the stress conditions is accompanied by an activation of NRF2-signaling pathway [39, 40].

The observed fullerene-induced decrease of ROS level in serum-starving HELFs considerably enhances the cell's viability. For instance, a 1.5-fold increase in the cell count was observed when F-828 was introduced in the culture of serum-starving HELFs in concentrations of 0.2–0.5 *µ*M. This effect is caused by both enhancement of proliferative activity of the cells and lowering the level of the cell death. An exposure to F-828 at concentrations of 0.2 and 0.25 *µ*M showed the minimum double-strand DNA break occurrence and the lowest cell mortality. It is notable that these positive effects were less pronounced at all the other concentrations of the fullerene derivative F-828 we used in the tests. Considerable improvement in the viability and survivability of the serum-starving HELFs in the presence of fullerene F-828 was accompanied by even more pronounced effects such as a strong increase in the activity.

It is important to note that elevated survival rate of serum-starving HELFs can be observed at F-828 concentrations from 0.2 up to 0.5 *µ*M. At these fullerene concentrations, the ROS level found in serum-starving HELFs decreased down to the level registered in control cells (2% FBS). More effective ROS scavenging at a concentration of F-828 higher than 0.5 *µ*M resulted in an increased cell death rate in comparison with the rate found in serum-starving HELFs without fullerene. A decrease in ROS level is accompanied by a decrease in the level of NF-*κ*B factor and an increase in the number of DSBs and in the dead cell count. The findings suggest that even in oxidative stress conditions surplus ROS elimination may be associated with a decrease in the cell survival rate. The harmful effect could be due to a different, ROS-unrelated mechanism(s). This interesting fact warrants further investigation.

## Additional Points

*Highlights*

(i) The influence of a water-soluble [60] fullerene derivative (F-828) used in concentrations of 0.1 *µ*M to 1 *µ*M on serum-starving human embryo lung diploid fibroblasts (HELFs) was studied.
(ii) Within a range of concentrations between 0.2 *µ*M and 1 *µ*M F-828 induced a decrease in the ROS level in HELFs.
(iii) In concentrations of 0.2–0.25 *µ*M F-828 enhanced proliferation of HELFs and decreased the DSB rate and apoptosis level.
(iv) F-828 strongly stimulated NF-*κ*B activity in serum-starving HELFs.
(v) F-828 repressed NRF2 activity and NOX4 expression in serum-starving HELFs.
(vi) In concentrations higher than 0.5 *µ*M F-828 induced excessive ROS scavenging that resulted in an increased cell death rate as compared to the rate in serum-starving (stressed) HELFs without fullerene.

## References

[1] Bakry R., Vallant R. M., Najam-ul-Haq M. (2007). Medicinal applications of fullerenes. *International Journal of Nanomedicine*.

[2] Qiao R., Roberts A. P., Mount A. S., Klaine S. J., Ke P. C. (2007). Translocation of C_60_ and its derivatives across a lipid bilayer. *Nano Letters*.

[3] Markovic Z., Trajkovic V. (2008). Biomedical potential of the reactive oxygen species generation and quenching by fullerenes (C60). *Biomaterials*.

[4] Lao F., Chen L., Li W. (2009). Fullerene nanoparticles selectively enter oxidation-damaged cerebral microvessel endothelial cells inhibit JNK-related apoptosis. *ACS Nano*.

[5] Ali S. S., Hardt J. I., Dugan L. L. (2008). SOD Activity of carboxyfullerenes predicts their neuroprotective efficacy: a structure-activity study. *Nanomedicine*.

[6] Lao F., Li W., Han D. (2009). Fullerene derivatives protect endothelial cells against NO-induced damage. *Nanotechnology*.

[7] Misirkic M. S., Todorovic-Markovic B. M., Vucicevic L. M. (2009). The protection of cells from nitric oxide-mediated apoptotic death by mechanochemically synthesized fullerene (C_60_) nanoparticles. *Biomaterials*.

[8] Yin J.-J., Lao F., Fu P. P. (2009). The scavenging of reactive oxygen species and the potential for cell protection by functionalized fullerene materials. *Biomaterials*.

[9] Ye S., Chen M., Jiang Y. (2014). Polyhydroxylated fullerene attenuates oxidative stress-induced apoptosis via a fortifying Nrf2-regulated cellular antioxidant defence system. *International Journal of Nanomedicine*.

[10] Kensler T. W., Wakabayashi N., Biswal S. (2007). Cell survival responses to environmental stresses via the Keap1-Nrf2-ARE pathway. *Annual Review of Pharmacology and Toxicology*.

[11] Ye S., Zhou T., Cheng K. (2015). Carboxylic acid fullerene (C_60_) derivatives attenuated neuroinflammatory responses by modulating mitochondrial dynamics. *Nanoscale Research Letters*.

[12] Gloire G., Legrand-Poels S., Piette J. (2006). NF-*κ*B activation by reactive oxygen species: fifteen years later. *Biochemical Pharmacology*.

[13] Hayden M. S., Ghosh S. (2012). NF-*κ*B, the first quarter-century: remarkable progress and outstanding questions. *Genes & Development*.

[14] Pedruzzi L. M., Stockler-Pinto M. B., Leite M., Mafra D. (2012). Nrf2-keap1 system versus NF-*κ*B: the good and the evil in chronic kidney disease?. *Biochimie*.

[15] Wakabayashi N., Slocum S. L., Skoko J. J., Shin S., Kensler T. W. (2010). When NRF2 talks, who's listening?. *Antioxidants & Redox Signaling*.

[16] Li W., Khor T. O., Xu C. (2008). Activation of Nrf2-antioxidant signaling attenuates NF*κ*B-inflammatory response and elicits apoptosis. *Biochemical Pharmacology*.

[17] Kostyuk S. V., Tabakov V. J., Chestkov V. V. (2013). Oxidized DNA induces an adaptive response in human fibroblasts. *Mutation Research/Fundamental and Molecular Mechanisms of Mutagenesis*.

[18] Kornev A. B., Khakina E. A., Troyanov S. I. (2012). Facile preparation of amine and amino acid adducts of [60]fullerene using chlorofullerene C_60_Cl_6_ as a precursor. *Chemical Communications*.

[19] Troshina O. A., Troshin P. A., Peregudov A. S., Kozlovskiy V. I., Balzarini J., Lyubovskaya R. N. (2007). Chlorofullerene C_60_C_l6_: a precursor for straightforward preparation of highly water-soluble polycarboxylic fullerene derivatives active against HIV. *Organic and Biomolecular Chemistry*.

[20] Podchernyaeva R. I. A., Baklanova O. V., Glushchenko L. A. (2010). Influenza virus reproduction in the MDCK cells adapted to growth in serum-free Hybris-2 medium. *Voprosy Virusologii*.

[21] Santos S. M., Dinis A. M., Peixoto F., Ferreira L., Jurado A. S., Videira R. A. (2014). Interaction of fullerene nanoparticles with biomembranes: from the partition in lipid membranes to effects on mitochondrial bioenergetics. *Toxicological Sciences*.

[22] Dumas J. J., Merithew E., Sudharshan E. (2001). Multivalent endosome targeting by homodimeric EEA1. *Molecular Cell*.

[23] Guillaud P., du Manoir S., Seigneurin D. (1989). Quantification and topographical description of Ki-67 antibody labelling during the cell cycle of normal fibroblastic (MRC-5) and mammary tumour cell lines (MCF-7). *Analytical Cellular Pathology*.

[24] Naryzhny S. N. (2008). Proliferating cell nuclear antigen: a proteomics view. *Cellular and Molecular Life Sciences*.

[25] Li L., Tan J., Miao Y., Lei P., Zhang Q. (2015). ROS and autophagy: interactions and molecular regulatory mechanisms. *Cellular and Molecular Neurobiology*.

[26] Klionsky D. J., Abdelmohsen K., Abe A. (2016). Guidelines for the use and interpretation of assays for monitoring autophagy (3rd edition). *Autophagy*.

[27] Cave A. C., Brewer A. C., Narayanapanicker A. (2006). NADPH oxidases in cardiovascular health and disease. *Antioxidants and Redox Signaling*.

[28] Hecker L., Logsdon N. J., Kurundkar D. (2014). Reversal of persistent fibrosis in aging by targeting Nox4-Nrf2 redox imbalance. *Science Translational Medicine*.

[29] Nguyen T., Sherratt P. J., Nioi P., Yang C. S., Pickett C. B. (2005). Nrf2 controls constitutive and inducible expression of ARE-driven genes through a dynamic pathway involving nucleocytoplasmic shuttling by Keap1. *Journal of Biological Chemistry*.

[30] Dreger H., Westphal K., Weller A. (2009). Nrf2-dependent upregulation of antioxidative enzymes: a novel pathway for proteasome inhibitor-mediated cardioprotection. *Cardiovascular Research*.

[31] Kovac S., Angelova P. R., Holmström K. M., Zhang Y., Dinkova-Kostova A. T., Abramov A. Y. (2015). Nrf2 regulates ROS production by mitochondria and NADPH oxidase. *Biochimica et Biophysica Acta (BBA)—General Subjects*.

[32] Pendyala S., Moitra J., Kalari S. (2011). Nrf2 regulates hyperoxia-induced Nox4 expression in human lung endothelium: identification of functional antioxidant response elements on the Nox4 promoter. *Free Radical Biology and Medicine*.

[33] Brewer A. C., Murray T. V. A., Arno M. (2011). Nox4 regulates Nrf2 and glutathione redox in cardiomyocytes in vivo. *Free Radical Biology and Medicine*.

[34] Löbrich M., Shibata A., Beucher A. (2010). *γ*H2AX foci analysis for monitoring DNA double-strand break repair: strengths, limitations and optimization. *Cell Cycle*.

[35] Kohno T., Kubo Y., Yasui K. (2012). Serum starvation activates NF-*κ*B through G protein *β*2 subunit-mediated signal. *DNA and Cell Biology*.

[36] Wang D., Baldwin A. S. (1998). Activation of nuclear factor-*κ*B-dependent transcription by tumor necrosis factor-*α* is mediated through phosphorylation of RelA/p65 on serine 529. *Journal of Biological Chemistry*.

[37] Kuznetsov A. V., Kehrer I., Kozlov A. V. (2011). Mitochondrial ROS production under cellular stress: comparison of different detection methods. *Analytical and Bioanalytical Chemistry*.

[38] Liu C., DeRoo E. P., Stecyk C., Wolsey M., Szuchnicki M., Hagos E. G. (2015). Impaired autophagy in mouse embryonic fibroblasts null for Krüppel-like Factor 4 promotes DNA damage and increases apoptosis upon serum starvation. *Molecular Cancer*.

[39] Stępkowski T. M., Kruszewski M. K. (2011). Molecular cross-talk between the NRF2/KEAP1 signaling pathway, autophagy, and apoptosis. *Free Radical Biology and Medicine*.

[40] Chen N., Wu L., Yuan H., Wang J. (2015). ROS/autophagy/Nrf2 pathway mediated low-dose radiation induced radio-resistance in human lung adenocarcinoma A549 cell. *International Journal of Biological Sciences*.

